# The Gut Microbiota: a Novel Player in the Pathogenesis of Uterine Fibroids

**DOI:** 10.1007/s43032-023-01289-7

**Published:** 2023-07-07

**Authors:** Vineetha K K, Rajeshwari G Bhat, Bhamini Krishna Rao, Archana P R

**Affiliations:** 1 Department of Obstetrics and Gynecology, Melaka Manipal Medical College, Manipal Academy of Higher Education, Manipal, Karnataka India; 2 Department of Physiotherapy, Manipal College of Health Professions, Manipal Academy of Higher Education, Manipal, Karnataka India; 3 Department of Basic Medical Sciences, Manipal Academy of Higher Education, Manipal, Karnataka India

**Keywords:** Estrogen, Gut microbiota, Lipopolysaccharides, Lifestyle, Short chain fatty acids, Uterine fibroid

## Abstract

Uterine fibroid is a common gynecological disorder that affects women of reproductive age and has emerged as a major public health concern. The symptoms have a negative influence on both their physical health and quality of life. The cost of treatment has a significant impact on the disease’s burden. Even though its origin is uncertain, estrogen is thought to be a key player in fibroid pathophysiology. Many theories, including those based on genetic and environmental factors, explain what causes hyper-estrogenic condition in fibroid patients. One such possibility that is currently being explored is the hypothesis that an altered gut microbiome can contribute to the development of diseases characterized by estrogen dominance. Gut dysbiosis is often a “hot area” in the health sciences. According to a recent study, uterine fibroid patients have altered gut microbiome. A variety of risk factors influence both fibroid development and gut homeostasis. Diet, lifestyle, physical activity, and environmental contaminants have an impact on estrogen and the gut flora. A better understanding of uterine fibroids’ pathophysiology is required to develop effective preventative and treatment options. A few ways by which the gut microbiota contributes to UF include estrogen, impaired immune function, inflammation, and altered gut metabolites. Therefore, in the future, while treating fibroid patients, various strategies to deal with changes in the gut flora may be advantageous. For developing suggestions for clinical diagnosis and therapy, we reviewed the literature on the relationship between uterine fibroids and the gut microbiota.

## Introduction

In recent years, leiomyomas, also known as uterine fibroids (UF), a prevalent gynecologic condition, have grown to be a major health issue [1]. What makes this a complex condition? It is often diagnosed incidentally. It varies in size and location. Sometimes patients are asymptomatic, despite the presence of a large fibroid, at the same time, there may be another patient with small fibroids who is experiencing significant symptoms like heavy menstrual bleeding and/or pelvic pain. Why do certain fibroids grow rapidly while others do not? Why does it recur to some women not in others? Are we overlooking the disease's diversity and heterogeneity? Are we unnecessarily making women apprehensive by diagnosing fibroids in many who otherwise have never known the presence of asymptomatic fibroids? Are present diagnostic and therapeutic treatment choices being adequate or are they exaggerating to tackle these puzzles?

According to many global statistics UFs are found in 20–80% of reproductive-age women [1]. Furthermore, around 30% [2] of them may experience severe symptoms, such as heavy menstrual bleeding and pelvic pain which affect their quality of life [3]. The cost of treatment contributes significantly to the disease burden [4]. Both medical management and surgical management, which are currently accessible therapeutic options, have variable adverse effects. Drug-based therapy can provide only symptomatic relief. When it comes to surgical alternatives, younger women's fertility and pregnancy outcomes may be compromised [5]. If we choose myomectomy over hysterectomy in such circumstances, the odds of fibroids recurrence are considerable. Hysterectomy (abdominal, vaginal and laparoscopic) is the most common surgical procedure; however, it has long-term complications such as pelvic floor dysfunction, the risk of fracture, etc. [6, 7]. Novel minimally invasive procedures such as operative hysteroscopy, uterine artery embolization (UAE), and high intensity focused ultrasound (MRgFUS), and laparoscopic myomectomy are now available only in tertiary care facilities with large setups [8, 9]. However, the lack of these facilities in the vast public health-care system limits their accessibility to the world's common middle and lower classes [10].

Numerous factors, including genetic, hormonal, immunologic, environmental, and lifestyle factors, might contribute to fibroids’ growth and development [11]. A greater understanding of diverse pathogenesis pathways is required to enable effective prevention and management measures to lessen the health, economic, social, and psychological impact of these tumors [12]. These facts lead one to think for a more personalized or preventive therapeutic strategy for the management of the condition.

## Evidence of Estrogen Dominance in UF Patients

The extracellular matrix (ECM) and smooth muscle cells, as well as a considerable number of fibroblasts, make up much of the fibroid tissue. The ECM is secreted by fibroblasts, which also provide fibroid cells with a framework for survival and nutritional support [13]. Even though the etiology is still unclear, there are many factors explained as the cause for fibroid development. Family history, hormonal changes, stress are some of those factors. Several experimental, epidemiological, and clinical studies have revealed that ovarian hormones such as estradiol and progesterone are involved in the development and pathophysiology of UF [14].

The prevalence of this disease among women of reproductive age, as well as its absence/reduced prevalence before and after puberty, provide the most convincing proof that estrogen has a role in UF [2, 15, 16]. Estradiol contributes to the development of fibroids, as evidenced by the peak incidence in the last premenopausal years with anovulatory cycles when there is high estrogen and a drop in progesterone levels [16]. Women with elevated levels of estradiol and testosterone were found to have an elevated incidence of UF, according to Wong et al. in 2016 [15]. The pathogenesis of UF is aided by estrogen's ability to increase the gene expression of many growth factors, collagens, and estrogen and progesterone receptors (ER, PR). The number of ERs and PRs in the cytoplasm of fibroids was significantly higher than that of normal myometrium, and their estrogen affinity was also higher [16, 17]. The susceptibility to UFs was elevated by a specific ER polymorphism. The circulating estrogens produced by ovarian steroidogenesis cause fibroids tissues to respond. By converting androgens locally, the aromatase gene's prominent expressions in UF also contribute to the hyper-estrogenic condition [16, 17]. The two nuclear estrogen receptors (ERs), ER-α and ER-β, which are present in both myometrial and fibroid tissues, coordinate the actions of estrogen. In addition to these hypotheses, research has shown that ER- polymorphism increases fibroid susceptibility. Although ovarian steroidogenesis is the main source of estrogen in the blood, aromatase enzymes locally convert androgens to estrogens, which increases the amount of estrogen in circulation. Comparing fibroids to healthy myometrium, it was found that estrogen levels rose along with aromatase and 17-hydroxysteroid dehydrogenase (-HSD) type-1 levels. Progesterone plays a role in the pathophysiology of UF in addition to estrogen and aromatase enzymes. Leiomyoma cells require progesterone to develop and multiply. Meanwhile, estradiol increases the availability of progesterone receptors, enhancing the tissue sensitivity to progesterone [13, 15].

## Gut Microbiota

The collective genomes of the microbiota that inhabit the human body, including viruses, bacteria, eukaryotes, protozoa, and archaea, compose the human microbiome [18, 19]. Five bacterial phyla dominate the gut microbiome: phylum Firmicutes (*Clostridium, Lactobacillus, Eubacterium, Ruminococcus, Butyrivibrio, Anaerostipes, Roseburia, Faecalibacterium,* etc; Gram-positive species), phylum Bacteroidetes (*Bacteroides, Porphyromonas, Prevotella,* etc; Gram-negative species), phylum Actinobacteria (*Bifidobacterium;*Gram-positive species), phylum Proteobacteria (*Escherichia,Klebsiella, Enterobacter;*Gram-negative species*),* and phylum Verrucomicrobia (*Akkermansia,* etc.; Gram-negative species) [19, 20]. Firmicutes and Bacteroidetes make up around 90% of the gut microbiota, with Actinobacteria and Proteobacteria making up the remaining 10%. The least common is Verrucomicrobia [21, 22].

The intricate connections between the gut microbiota and human health, particularly with regard to immune responses and dietary metabolism, have been extensively researched [23, 24]. Changes in microbial interactions, on the other hand, affect the host’s local immune system, altered gut metabolites, and the functioning of the intestinal barrier. Changes that disturb the intestinal homeostasis led to the emergence of a variety of human diseases, such as malignant tumors, chronic infectious diseases, gastrointestinal disorders, and metabolic disorders [25, 26]. New therapies and interventions focus on the gut flora to improve health.

## Factors Influencing Microbiota Composition

### Age

The first few years of life are when the microbiome is most colonized [27]. During and immediately after delivery, newborns are exposed to maternal and ambient microorganisms, which initiate the development of the gut microbiome [27, 28]. Following weaning, the gut microbiota establishes itself securely and creates a lifetime microbiome signature in healthy individuals [27]. Although the makeup and functionality of the healthy adult gut microbiome are often constant over extended periods of time (normally >65 years), ageing has a substantial impact on these factors [29, 30]. A considerable imbalance in the main phyla, such as the anaerobic Firmicutes and Bacteroidetes, as well as a wide variety of facultative organisms develops in the gut microbiota with age, impairing immune responses [27]. According to a study by Mariat D et al. [31] the ratio of Firmicutes to Bacteroidetes changed in older people consuming a Western-style diet, dropping from 10.9 in adults (25–45 years) to 0.6 in the elderly cohort (70–90 years), which is like the ratio of 0.4 in infants (three weeks to 10 months old). In a study by Zhao et al. [30], the richness of the gut microbiota at the gene, species, and genus levels was significantly reduced in postmenopausal women as compared to premenopausal women. The Firmicutes to Bacteroidetes ratio was changed in postmenopausal women [30].

### Genetics

The number of certain bacteria within the gut microbiota is impacted by the host’s genetic makeup [27, 32]. According to a study, monozygotic twins’ gut microbiota are more comparable than that of dizygotic twins, and the microbiota communities of family members are more alike than those of people who are not related [32].

### Diet

Diet and the gut microbiome have been extensively investigated [33]. To date, findings suggest that long-term nutrition has an extremely ample influence on gut microbiome composition [34]. Changes in the diet increase the availability of certain nutrients, influencing which bacteria dominate and contributing to the dynamic nature of the microbiome. For example, *Prevotella* species are connected to plant-based diets, and *Bacteroides* species tend to predominate in the GI tract of people who consume animal protein [35]. A notable high abundance of *Bifidobacterium,* a genus known to produce significant numbers of starch-metabolizing enzymes, is found in the enterotypes of Asian cultures that consume substantial amounts of starch-rich foods like rice [36]. Comprehensive research has indicated that the Western diet, which is rich in saturated fats but short in unsaturated fats, is positively associated with anaerobic bacteria and specific species such as *Bacteroides and Bilophila* [37].

### Antibiotic use

Depending on the type and duration of use, the host microbiota may rapidly alter its structure when exposed to antibiotics [38]. The use of antibiotics reduces the gut microbial diversity, including the loss of several key taxa, resulting in metabolic changes, a greater susceptibility to colonization, and the development of bacterial antibiotic resistance [38].

## Gut Dysbiosis

An imbalance in the gut microbiota termed gut dysbiosis may result from the translocation of colonic bacteria, an increase in small bowel bacteria, a shift in the relative ratio of beneficial to pathogenic microbes, or any combination of these factors [24, 39, 40]. There is mounting evidence linking gut microbial dysbiosis to the etiology of numerous disorders, including irritable bowel syndrome (IBS), Crohn disease, obesity, etc. [23, 24, 41, 42]. A member of the phylum Proteobacteria, *Helicobacter pylori* (*H. pylori*) is a Gram-negative bacterium that can inhabit the stomach [43]. The most common modes of transmission are oral-oral, fecal-oral, and environmental and food contamination [44]. However, a few investigations have shown that these bacteria may be transmitted through sexual contact [44, 45]. *H. pylori* infection affects over fifty per cent of the world's population, and disturbing gut homeostasis has also been linked to a number of metabolic and gastrointestinal conditions [43, 46]. According to recent research, *H. pylori* infection affects the harmony of commensal bacterial species in the gastric mucosa as well as the microbiome of the human gut [47]. Proteobacteria, especially *Epsilon-proteobacteria*, *Spirochetes,* and *Acidobacteria* were all shown to be predominant in the microbiota of *H. pylori* positive people, while Actinobacteria, Bacteroidetes, and Firmicutes were found to be less prevalent [47].

More and more evidence point to a connection between a dysbiotic gut and common reproductive disorders such polycystic ovary syndrome (PCOS), infertility, endometriosis, and/or dysregulated ovarian processes (Table 1).

**Table 1:** Studies on gut dysbiosis among subjects with gynecological disorders

Study	Hypothesis tested	Design &subjects	Results
Yanjie Guo, 2016 [48]	The gut microbiota's role in the etiology and treatment of PCOS in a letrozole-induced rat model	Alterations in the estrous cycle, hormone levels, ovarian morphology, and gut microbiome in 18 rats with PCOS and 18 normal control rats.	Abnormal estrous cycles with increased androgen synthesis in PCOS rats. *Prevotella* was higher and *Lactobacillus, Ruminococcus,* and *Clostridium* were lower in the PCOS group than in the control group, but there were no statistically significant differences for *Bifidobacterium, Escherichia coli, Enterococcus,* or *Bacteroides.*
Rui Liu, 2017 [49]	Gut dysbiosis is associated with clinical parameters in PCOS patients	Anthropometric and metabolic measurements and gut microbiome sequencing among 33 PCOS patients and 15 controls (both obese and non-obese)	PCOS patients’ (both obese and non-obese) gut microbiota shares a similar compositional dysbiosis with that of obesity. The *Clostridial* species were reduced, but *Bacteroides spp.* were increased in both the PCOS and obese cohorts.
Agnes Svensson, 2021[50]	The potential links between endometriosis and gut microbiota	16s rRNA sequencing among 66 endometriosis patients and 198 control subjects	A significantly higher alpha and beta diversities of the microbiota were present in controls than in patients. Between patients and controls, there were significant differences in the abundance of 12 bacteria from the classes *Bacilli, Bacteroidia, Clostridia, Coriobacteria, and Gammaproteobacter*.
Liujing Huang, 2021[51]	Correlation between endometriosis patients' gut microbiota, cervical mucus, and peritoneal fluid microbiome	The microbiome of 21 endometriosis patients and 20 control was sequenced using 16 S rRNA from feces, cervical mucus, and peritoneal fluid.	Endometriosis patients have different microbial communities, particularly in the feces and peritoneal fluid. Ten taxa, including *Clostridium Clostridiales, Lachnospiraceae Ruminococcus,* and *Ruminococcaceae Ruminococcus,* were considerably decreased in the fecal microbiota of patients compared to controls, whereas two taxa, including *Eggerthella lenta* and *Eubacterium dolichum*, were significantly enriched.

*PCOS* polycystic ovarian syndrome

## Changes in Gut Microbiome in UF Patients

The gut flora of UF patients, however, is not the subject of many reports [52–55]. According to the most current research by Mao et al. [53], patients with UF showed decreased gut microbial diversity and less connect in their community networks than controls. They found that various bacterial phyla, including Firmicutes, Proteobacteria, Actinobacteria, and Verrucomicrobia, have undergone considerable alterations in fibroid patients. These results indicate a possible link between gut dysbiosis and the potential development of UF. In a case-control study, the effects of trans-abdominal hysterectomy on the gut flora and variations in follicle-stimulating hormone (FSH), estradiol (E2), and anti-Mullerian hormone (AMH) in UF patients were investigated [53]. The study reported that trans-abdominal hysterectomy changed ovarian function, resulting in lower E2 and AMH and higher FSH. The gut microbiome composition shifted when estrogen levels dropped, with more Proteobacteria and Firmicutes with lesser Bacteroidetes. [52, 54].

**Fig. 1:**
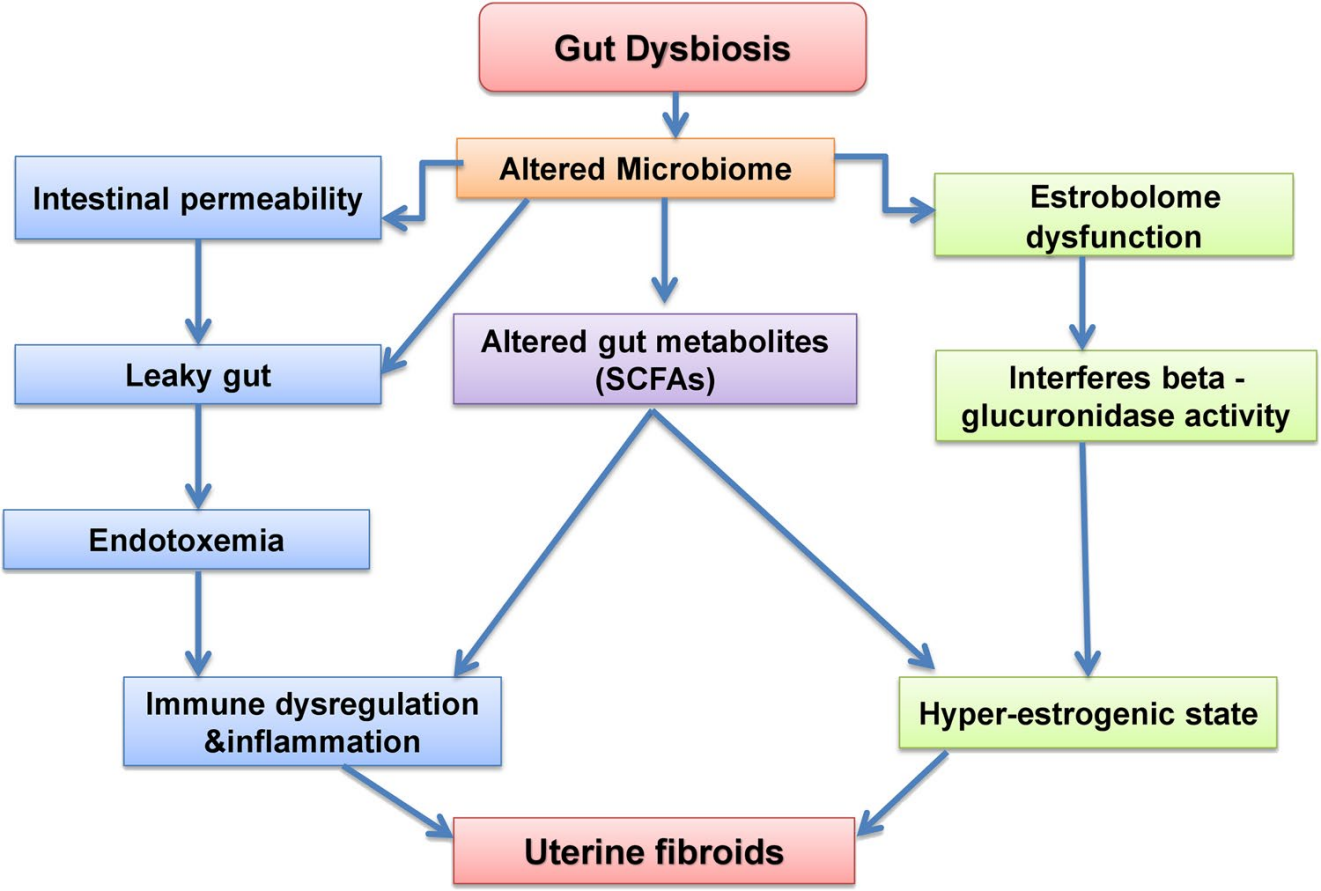
Possible mechanisms that could link gut dysbiosis and the development of uterine fibroids [2, 15, 53, 54, 56–63]. A dysbiotic gut results in an altered microbiome which triggers the following pathways i) interferes with the estrobolome functioning and results in hyperestrogenic state leading to uterine fibroids, ii) altered levels of gut metabolites such as short chain fatty acids (SCFA) which triggers immune dysregulation and induce inflammation and also leads to hyperestrogenic state, iii) interferes the gut permeability leading to a leaky gut and endotoxemia which could dysregulate the immune function

Numerous mechanisms might be active in this interaction (Fig. 1). Unhealthy nutrition and sex hormones have been recognized as the two main risk factors for UF. Unhealthy eating habits can lead to dysbiosis in the gut [37]. The gut microbiota may also influence sex hormone levels as a result of the interactions with the immune system, chronic inflammation, and its metabolites [64].

## Estrobolomes and Endobolomes

Estrobolomes, a group of bacteria that includes numerous species including *Eubacterium lentum*, *Bacteroides* sp., *Bifidobacterium* sp., *Streptococcus* sp., etc. that may modify and metabolize estrogen, make up the human gut microbiome [65]. Bacterial -glucuronidases (BG) catalyze the hydrolysis of endogenous -glucuronides produced in the liver and exogenous -glucuronides present in the diet, like complex polysaccharide [56, 66].

The endobolome is a collection of genes and pathways in the gut microbiota that involve in the metabolism of steroid hormones and endocrine disrupting chemicals (EDCs) [67]. The Endocrine Society recently defined the Endocrine Disrupting Chemicals (EDC) as “an exogenous [non-natural] chemical, or mixture of chemicals, which interferes with any aspect of hormone action.” Dioxins, pesticides, pyrethroids, polychlorinated biphenyls (PCBs), flame retardants, antibacterials like triclosan, compounds originating from plants like phytoestrogens, and plasticizers like phthalates and bisphenol A (BPA) are some of them [37, 68–70].

After hepatic glucuronidation, bile excretes many metabolites, steroid hormones, and xenobiotics into the intestinal tract. The intestinal bacterial β-glucuronidases' helps in elimination of glucuronic acid from conjugated substrates (deconjugation) and encourages reabsorption of the corresponding aglycones into the enterohepatic circulation. Different bacterial ß-glucuronidase genes have been identified in the human gut microbiome [71, 72]. The bacterial phyla Bacteroidetes have a large distribution of the BG gene. Genes for β-glucuronidase (GUS) are widely found in Firmicutes [73]. The product of these bacterial genes is capable of metabolizing estrogens by secreting estrogen degrading enzyme β – glucuronidase and steroid hormone synthesizing enzyme hydroxysteroid dehydrogenases (HSDs) [74] allowing it to bind to estrogen receptors and producing the physiological outcomes that follow [75]. The only estrogen that is biologically active is the free, unbound form. The majority of conjugated estrogen is bound by a liver-produced glycoprotein called sex hormone binding globulin (SHBG) [76].

Gut bacteria can change the expression of genes involved in estrogen metabolism. *Escherichia coli*, *Bacteroides species, and Clostridium perfringens* are examples of bacterial species found in estrobolome that have the enzyme β-glucuronidase (GUS), which deconjugates conjugated estrogens by cleaving the glucuronic acid and releasing free estrogens that are then reabsorbed into the bloodstream and repeatedly circulated through the enterohepatic system [65, 76–78]. According to Baker et al. [76], it is these “active” deconjugated and free estrogens that enter the bloodstream and act on estrogen receptor alpha (ER-α) and estrogen receptor beta (ER-β).

EDC exposure may cause significant alterations in the gut flora, leading to extensive abnormalities in multiple systems [79]. This can be accomplished by altering microbial diversity and causing dysbiosis, by metabolizing xenobiotics directly and indirectly, by interfering with microbial enzyme function, and by changing microbial diversity [80]. Both BPA and ethinyl-estradiol exposure have been shown to increase Bifidobacterium spp. in mice, which can cause metabolic disorders [81]; whereas methylparaben, diethyl phthalate, and triclosan (or their mixture) exposure changes the ratio of Bacteroidetes to Firmicutes in rats [82], which is an important indicator of gut dysbiosis.

Another potential enzymatic activity of the estrobolome is dehydrogenation [83, 84]. According to reports from an observational study, women who had a diverse microbiome reported higher urine ratios of hydroxylated estrogen metabolites to parent estrogen [85]. These observational results lend credence to the idea that variations in gut microbial diversity are related to variations in estrogen metabolism and levels. Newly diagnosed postmenopausal breast cancer patients had a less varied microbiome, according to Goedert and his colleagues [86]. However, the patients also reported higher urine estrogen levels than in the control group, which was unrelated to the diversity of the microbiota. Apart from these facts, an in-vitro study performed by Lu et al. [57] had demonstrated butyrate mediated regulation of estradiol and progesterone via the cAMP signaling pathway.

## Gut Dysbiosis Induced Immune Dysregulation and Inflammation

Constant connections between the gut microbiota and the host’s innate and adaptive immune systems are essential to maintain intestinal homeostasis and prevent inflammation [87]. Numerous species of gut microbes break down proteins and complex carbohydrates to produce a vast array of metabolites that engage in the interaction between immune cells and the gut epithelium. Additionally, these metabolites have anti-inflammatory and immuno-modulatory properties, production of vitamins, inhibit and activate certain immune system responses, and support the integrity of the gut wall by maintaining the gut epithelium [87, 88]. The distinct functions mentioned above can become dysregulated in a dysbiotic state, which can then contribute to the emergence of inflammatory and autoimmune diseases [88–90]. The host's inflammatory states may be controlled by the gut microbiota and its metabolites [91, 92].

It has been hypothesized that UF development is influenced by a persistently active inflammatory immune system [58, 59] (Fig. 1). Numerous connections have been discovered between the increased prevalence of UF inflammatory mediator gene polymorphisms, including interleukin (IL)-1 [93], IL-4 [94], TNF [94, 95], IL-6 [96], IL-10 [97], and IL-12b receptor [98]. A recent study in a Chinese population identified that, compared to healthy controls, patients with UF had significantly higher levels of circulating CD4+CD8+ T cells, regulatory T (Treg, CD4+) cells, and T follicular helper (Tfh) cells, while lower levels of natural killer (NK) and delta gamma T cells (CD4 ,CD8) [54, 99].

## Short-Chain Fatty Acid Metabolism

The most frequent SCFAs are acetate (C2), propionate (C3), and butyrate (C4), which are carboxylic acids with aliphatic tails of 1-6 carbons formed through anaerobic fermentation of dietary fibers (DF) in the colon [100]. The gram-positive Firmicutes and gram-negative Bacteroidetes are the two phyla that are most common in the intestine [20]. Bacteroidetes mostly form butyrate and acetate. Though it can also emerge from the metabolism of organic acids and amino acids, glycolysis is the main source of butyrate and propionate synthesis in the gut [101]. Acetate, a predominant SCFA found in human gut, is also produced from acetyl-CoA, which is derived by glycolysis, and it can be transformed into butyrate by the enzyme butyryl-CoA: acetyl-CoA transferase [60, 102]. These SCFAs can be eliminated through feces or can be reabsorbed by the gut [60, 101]. The reabsorbed SCFA can participate in a number of physiological functions. Butyrate serves as the colonocytes' major energy source, aids in maintaining the gut barrier, and prevents the entry of powerful inflammatory signals like lipopolysaccharides (LPS) [102, 103]. SCFA in the intestinal mucosa induces intracellular or extracellular processes that have positive effects on immune cells and intestinal epithelial cells (IECs) [102–104] (Fig. 1). SCFA may diffuse passively through the cell membrane [103, 104]. Additionally, SCFAs influence peripheral immunological responses by inducing T-regulatory cell differentiation [105]. Recent research has identified fecal SCFA as a useful, trustworthy, and non-invasive biomarker for disorders connected to the gut microbiota, like inflammatory bowel disease (IBD) [60, 106]. The interaction of SCFAs with free fatty acid receptor-2 (FFAR-2) and FFAR-3, which are present in tissues such the intestines, fat, skeletal muscle, liver, immune system, and neurological system, might inhibit the production of appetite-stimulating hormone (ASH) by the gastric mucosa [107]. Growth hormone releasing hormone (GnRH) and sex hormone release are both prevented by ASH, in addition to the conversion of sex hormones [107]. According to Docanto et al. [61], ASH can prevent androgen from converting into estrogen by suppressing the expression of the aromatase CYP19A1 in adipo-stromal cells. A disturbed (dysbiosis) and unhealthy gut flora could influence the SCFAs concentrations [39, 40]

## Intestinal Permeability and Lipopolysaccharide Metabolism

Numerous gastrointestinal and non-gastrointestinal illnesses have been linked to increased intestinal permeability [62, 108]. The cellular bypass pathway’s initial line of defense, intestinal mucosal tight junctions are found at the top and edge of mucosal cell membranes and regulate the openings of intercellular pathways [109]. Intercellular tight junctions are complete normally, but they can lose their shape and function in pathological conditions such oxidative stress or inflammatory damage [108]. Intestinal permeability increases and tight junctions disintegrate as a result of the inflammatory cytokines TNF-α and INF-γ decreasing the expression of the tight junction proteins zonula occludens-1 (ZO-1) and occludin, according to studies [110].

Bacterial endotoxins, also called lipopolysaccharides (LPS), are a special part of the cell walls of gram-negative bacteria. The human intestine contains gram-negative bacteria called Bacteroides and Escherichia [62]. LPS can attach to immune cells’ Toll-like receptor 4 (TLR4) after entering the bloodstream by transmitting LPS-binding protein (LBP), CD14, and bone marrow differentiation factor (MD-2). This may activate signaling pathways downstream that encourage the expression of TNF-α, IL-6, and other molecules which can induce inflammation [111]. Under normal conditions, LPS cannot penetrate the intestinal epithelium and fails to promote the loss of intestinal epithelial cells at these physiologically significant levels, but it does influence the control of tight junction proteins. A dysbiotic gut changes intestinal permeability, and defective tight junction proteins allow LPS to enter the bloodstream [63, 111]. Numerous metabolic and inflammatory disorders can be brought on by this endotoxemia [63, 112] (Fig. 1).

## Lifestyle Changes for Management of UF and a Healthy Gut

Dietary habits and food ingredients may affect a person's likelihood of developing UF as well as gut health. Indeed, several hormone-related disorders may be exacerbated by contaminants found in foods including fruit, vegetables, and fish [113]. He et al. [114] conducted a case-control study that revealed fruit and vegetable consumption had a preventive effect on the etiology of fibroids in Chinese premenopausal women. Many pollutants impair women’s health by mimicking endogenous steroid hormones such as estrogen and progesterone and functioning as EDCs [68]. As per to a Chinese case-control study, urine and blood samples from fibroid patients had higher concentrations of phenolic environmental estrogens (bisphenol, nonylphenol, and octylphenol) than the healthy group [69]. The Endometriosis: Natural History, Diagnosis, and Outcomes (ENDO) study demonstrated a relationship between the diagnosis of UF and elevated blood levels of cadmium and lead as well as urine cobalt levels, further demonstrating the role of these trace elements in growth of the fibroid tumors [71].

Diet is a significant modifiable element which can alter the gut flora, although these modifications are transient [91]. Food contaminants and inadequate intakes of fruits, vegetables, and vitamin D are some of these contributing factors [113].

Vitamin D is a vital nutrient that is critical to human health [115]. Low concentrations of serum vitamin D (25(OH)D) have been linked with poor health outcomes [115]. Vitamin D’s significance in the etiology of UF has been studied [54, 55, 116]. Three substantial studies revealed that vitamin D levels in the sera of fibroid patients are significantly lower [117–119]. Recent research indicates that changes in vitamin D level or exposure can affect the composition of the gut microbiota [120, 121]. According to a study by Parul Sing et al. [121], vitamin D treatment considerably increased the gut microbiota in healthy women with vitamin D deficiency. This shift was particularly pronounced as the ratio of Bacteroidetes to Firmicutes increased. Vitamin D intake was found to be inversely correlated with *Prevotella* abundance and significantly linked with *Bacteroides*, both belonging to the phylum Bacteroidetes, in a cross-sectional study of healthy people [34]. Contrarily, Luthold et al. discovered that healthy people with higher reported vitamin D intake had lower levels of *Haemophilus* (phylum: Proteobacteria), *Veillonella* (phylum: Firmicutes), and *Prevotella* in their feces [120].

According to a study using animal models, the western diet has a detrimental effect on SCFA levels [122]. Consuming enough fermentable dietary fibers, or SCFA, appears to be clinically beneficial for preserving a healthy microbial flora [123]. Diet and bacterial environment can both affect β-glucuronidase activity in the gut. Diets high in fat or protein have been shown to boost fecal β-glucuronidase activity in healthy adults, although fiber consumption causes activity to decrease [124, 125]. Although long-term diets shape the composition of the gut flora, dietary changes can cause detectable shifts in some bacterial species within 24 hours [34]. A diet that is abundant in processed foods, sugar, and saturated fat contrasts sharply with the fiber-rich, plant-based diets of traditional cultures. Low gut microbial diversity and increased gut leakiness are characteristic of this diet, which may hasten the onset of metabolic syndrome and chronic diseases [126].

Women who regularly exercise and have a low BMI have been found to have a lower chance of developing fibroid. In a study by He et al., [114], the risk of fibroids was lower in women with moderate occupational intensity. According to the latest studies, exercise training altered the composition and function of gut flora independently [127, 128]. Individuals with obesity have a greater baseline ratio of Firmicutes to Bacteroidetes and reducing caloric consumption results in a lower Firmicutes to Bacteroidetes ratio [129, 130].

Chronic stress was correlated with fibroids, according to Vines et al. [131]. While there was a modest association between high levels of stress and fibroids in black women, white women with low to moderate levels of stress had a higher prevalence of UF than those with no stress. It can also affect colonic motor activity and modify variety of gut microbiome and its functions by reducing the number of commensal species *Lactobacillus* [132]. The increased inflammation that usually occurs with stress and depression promotes the growth of harmful bacteria, which promotes dysbiosis and a leaky gut [133]

## Probiotics and Prebiotics for Gut Health

Probiotics constitute beneficial active bacteria that colonize the human gut and modify the host flora in particular areas. Recent study has shown that probiotics are crucial to the gut microbiota and can help the host create a strong intestinal mucosa protective barrier and boost their immune systems by preventing the growth of pathogenic intestinal bacteria [134]. Probiotics also serve a positive role in the human body and have a special regulatory impact on the microecology of the gut. When probiotics were injected into obese animals, it was discovered that the gut flora’s Firmicutes had reduced and its Actinobacteria had increased. Intestinal flora dysbiosis in mice can be treated with probiotics to reduce inflammatory reactions [135]. Probiotic treatment describes the use of probiotics in the proper dosages to treat illness. By positively influencing different metabolic pathways, probiotics have been shown to have a potential impact in metabolic disorders [136, 137].

Non-digestible (oligo) saccharides known as prebiotics have been described as “selectively fermented substances that allow specific changes, both in the composition and/or activity of the gastrointestinal microbiota that bestow benefits upon host welfare and health.” Numerous research has looked into how prebiotics affect various metabolic illnesses. According to the studies, prebiotics enhance microbial fermentation, reduce appetite, and lower plasma glucose absorption post meals [138, 139].

## Conclusion

In conclusion, this review emphasizes recent developments in current research of the connection between UF and gut microbiota dysbiosis. Only a few studies have described the 16s rRNA gut microbiota sequencing in patients with uterine fibroid. However, recent research addresses the link between gut dysbiosis and other gynecological illnesses with a similar pathophysiology. More research must therefore focus on the intestinal flora of UF patients. However, we investigated the possibility that interactions between estrogen and gut microbiota dysbiosis could function as UF triggers. More research is needed to fully understand the processes at work. We only examined limited articles that examined the relationship between a hyper-estrogenic state and gut dysbiosis. Additionally, we looked at articles on several risk factors of UF and gut imbalance. These findings suggest additional research in this underrepresented area and hold the intriguing promise of identifying new targets for UF treatment and prevention. Finally, we looked at research on the effectiveness of probiotic and prebiotic therapies that affect the gut flora. To examine the changes in disease development and treatment outcomes, investigations involving prebiotic and probiotic therapy treatments are required within the UF population.

## Data Availability

Not applicable.
